# Sixteen Years of the Hospital Sunday Fund: Its Influence on the Metropolitan Medical Charities

**Published:** 1888-11-17

**Authors:** Sydney H. Waterlow

**Affiliations:** Treasurer of St. Bartholomew's Hospital


### SIXTEEN YEARS OF THE HOSPITAL SUNDAY FUND;

### ITS INFLUENCE ON THE MEDICAL CHARITIES OF THE METROPOLIS.

By SIR SYDNEY H. WATERLOW, Bart., Treasurer of St. Bartholomew's Hospital

*(where this paper was read on Thursday evening last).*

DURING the last few years a strong opinion has been expressed at public meetings, and in the leading daily and weekly papers, that the contributions collected each year on Hospital Sunday are far below the sum that ought to be subscribed by the inhabitants of this great metropolis of more than four millions of people, and are wholly insufficient as a proper subsidy towards the cost of maintaining the hospitals, dispensaries, convalescent homes, and other institutions making claims on the fund. Many persons have thought that the fund would be considerably increased if the public were better informed of the method adopted in the distribution or division of the receipts, in accordance with the "needs and merits" of the several institutions to be benefited. It may also be desirable to consider what influence the action of the Distribution Committee has exercised over the management and expenditure of these institutions.

It should be borne in mind that Hospital Sunday is quite a modern institution; so far as I am aware, the earliest suggestion in reference to it will be found in the *Midland Counties Herald* of October, 1859 (not thirty years ago), and the first Hospital Sunday collection was made in Birmingham on the 13th November in that year. The suggestion was contained in a letter written by the late Canon Miller, to whom we all owe so much in connexion with the establishment of Hospital Sunday in London. During the first fourteen years £56,000 was collected in Birmingham, while in London £432,976 was received in the same period. During the ten years from 1859 to 1869 the movement made very little, if any progress; but between 1869 and 1872, greatly owing to the attention called to the question by occasional articles and letters in the *Lancet*, a Sunday collection was made in many of our provincial towns, Manchester taking the lead, the collection there amounting to £16,244 in the first three years.

The proposal to set aside a Hospital Sunday for the metropolis was first considered at a meeting of the treasurers of the London hospitals, held at the London Tavern, November 21st, 1872, Mr. R. B. Martin in the chair. After a full discussion, it was resolved that Mr. Martin and Mr. Currie (now Sir Edmund Currie) should wait upon the Lord Mayor, who took up the proposal most earnestly; and from thenceforward the business of the fund has been conducted at the Mansion House, and each successive Lord Mayor, for sixteen years, has acted as president and treasurer of the fund, which has been dispensed with the greatest possible advantage to the sick poor of the metropolis. As the first President, and subsequently Vice-President, of the Fund, and Chairman of the Distribution Committee, I have naturally taken the deepest interest in the work. I believe that the movement has been most beneficial, apart from its usefulness as the most economical system of collecting and distributing money for our numerous medical charities. I was much drawn towards the work for two reasons:

*First.* That it is, I believe, a glorious thing for all of us to feel that, notwithstanding the great divergence in the creeds and forms of worship in the thousands of churches in our great metropolis, there is on *one* Sunday in each year a spirit of unity of religious thought and purpose among the worshippers in our churches and chapels, stimulating both clergy and laity alike to a healthy emulation in the great work of mercy and charity to the sick and suffering around us.

*Secondly.* That the examination and comparison of the accounts published yearly by the governing bodies of the several institutions claiming a share of the funds would induce a more careful management and economical expenditure.

That this expectation has been largely realised will, I think, be clearly evident to anyone who will take the trouble to look over the returns sent in annually to the Distribution Committee.

To further illustrate this latter reason, let me state that at the present time there are ten hospitals, seven convalescent homes and seven dispensaries that do not apply for any share of the funds. These may be classed under three heads:—

1. Those that are sufficiently supported by endowment or annual subscriptions and want no pecuniary help.
2. Some that, having been refused a share of the funds on the ground of bad and extravagant management, object to an examination of their accounts, and therefore cease to apply.
3. A few that are really merely private enterprises, founded and maintained for the profit and benefit of some member of the medical profession.

There are a large number of generous, warm-hearted, wealthy people desirous of helping the sick poor, and anxious to distinguish as clearly as possible the medical charities that ought, from those that ought not, to receive any share of their bounty. It may, perhaps, assist these good people and stimulate public subscriptions on Hospital Sunday, if I state, as briefly and closely as I can, the system adopted by the Committee of the Hospital Sunday Fund, in determining the "needs and merits" of each hospital and dispensary. It is a satisfaction to all donors to know what becomes of the money they contribute; how much is ultimately utilised in promoting the object they desire to assist, and how much of it is lost on the road in payment of the expenses of bazaars, public dinners, or other agencies by means of which the money is raised, and how much is spent in costly advertisements and salaries or commissions to professional collectors. Many will perhaps be surprised to learn that from the returns annually sent to the Mansion House it is evident, that, as a rule, of every 20*s.* given at bazaars or public dinners in aid of medical charities, a sum of from 4*s.* to 6*s.* has to be deducted for expenses of collection, and never reaches the suffering objects of those charities. In the case of charities frequently advertised in our daily and weekly journals, the percentage of deduction is very little less. Of the money received on Hospital Sunday less than 10*d.* in 20*s.* is spent in collection and distribution, including all salaries, advertising, etc. The total sum collected this year is £40,379 9*s.* 6*d.*, and for sixteen years £513,962. This amount includes all legacies. After deducting the expenses, it has been distributed in its entirety, the Council being of opinion that the sums collected each year are intended for the relief of the sick poor in that year.

The system adopted for the division of the money collected on Hospital Sunday may be thus briefly described:—

*Firstly.* A sum equal to 4 per cent. of the total receipts is set aside for the purchase of surgical appliances to be given away without cost and with comparatively little trouble to poor persons, who are recommended by the minister of any of the contributing congregations and are resident in his district, also for instruments which are certified as necessary by the surgeon of the hospital where the patient has been treated.

*Secondly.* All awards to hospitals, etc., are primarily based on the average total expenditure of each institution for the last three years, after deducting therefrom: 1. A sum equal to the income derived from endowments and realised property; 2. The amount received in legacies exceeding £100 each, unless such legacies have been necessarily spent to meet the current expenditure of the institution; 3. The amount of expenses of management. In every case the merits and pecuniary needs of the institution concerned are fully inquired into and considered by the Distribution Committee, and the award made is determined in accordance with the judgment of that Committee upon such merits and needs.

*Thirdly.* Each hospital or institution applying to participate is required to send in a report and balance-sheet for three successive years, and to fill up a printed form of receipts and expenditure for the same period. In order to arrive at the needs of each institution, the deductions previously stated are first made, and in the case of hospitals receiving payments from patients, and of provident dispensaries, such a proportion of the receipts from these sources of income is added as seems in each case justifiable. The sum thus arrived at forms the natural basis of the award, but this basis is augmented or diminished after a careful consideration, by the Committee, of the "merits" of each institution. The final decision on this point is very much influenced by the amount of work done in proportion to the money expended, the useful and practical character of the work; the proportion which charges for maintenance, nursing, and other sums, spent on patients, bears to the charges for administration and management, and by any other circumstances affecting the work of the institution then under consideration. As a result, the natural basis is sometimes greatly increased, sometimes diminished, and occasionally an award is altogether withheld, but in no case is it diminished or withheld without asking for an explanation. Great advantages have, I believe, resulted from the conferences at which these explanations are offered. Occasionally misapprehensions are rectified and the basis is restored; in other cases defects in management or extravagance in expenditure are pointed out, and when rectified in subsequent years the institution becomes entitled to an award.

The recipient charities having all to make a return of receipts and expenditure on a similar form, their accounts have by degrees been brought into a more uniform and intelligible shape, which greatly facilitates comparison. No item of unwise or improper expenditure can easily escape detection, and this is not without its public advantage.

It has been sometimes suggested that the money collected on Hospital Sunday should be divided according to the average number of beds occupied, the number of out-patients treated, and the cost per head in each case. This would, I think, in many cases be unfair, produce very unsatisfactory results, and lead to fallacious and misleading methods for the enumeration of the patients treated in any one year. There is no recognized principle for the classification and computation of patients, or for the division of the expenditure between in-patients and out-patients. Some hospitals treat a large number of out-patients suffering from very trivial ailments which can be summarily dealt with, while others only treat lingering and protracted cases, such as consumption, cancer, &c. This is the only way to account for the great difference in the returns of the cost of out-patients, the sum ranging from 10¾*d.* per patient at the Royal Free Hospital, to 25*s.* 1*d.* at the Cancer Hospital, Brompton, where the patients frequently attend for many months. The cost of the maintenance of in-patients does not vary to the same extent. Taking seven large general hospitals, viz., the London, Guy's, Charing Cross, King's College, Middlesex, University, and Westminster, we find that in the Middlesex Hospital, where there are nearly fifty beds for cancer cases, the cost is the highest, being 42*s.* per in-patient per week; and in the Westminster Hospital it is the lowest at 24*s.* 5*d.* If we turn to the special hospitals, excluding those for children, and the lying-in hospitals, the contrast is much the same, the cost per week varying from 28*s.* 2*d.* to 49*s.* per head per week. These figures are the sums returned by the hospital authorities, and the variation may, to some extent, arise from a different plan in calculating the expenses.

It is curious to note that the smaller the hospital and the more it claims speciality in its work, the greater is the proportion of its funds spent on management as compared with maintenance. The excessive cost of the management of small hospitals points to the evils arising of late years from the tendency to multiply the number of our medical charities instead of reducing them by amalgamation, coupled with a proper arrangement for dividing them over the thickly-populated districts of London. Instead of making any further attempts to establish new hospitals, our efforts ought to be directed to the collection of funds to fill the large number of empty beds in the hospitals already established, where the expenses of management would remain at nearly the same sum whether the beds are full or empty. That this course ought to be adopted is, I think, clearly evident from the fact that we have now 2,031 empty beds in seventy-one hospitals in various parts of the metropolis. It would not cost half as much money to maintain and treat patients in these beds as it would to provide the same number of beds in new hospitals.

The cost of the maintenance of patients has in most hospitals been considerably increased by the introduction of a system of skilled and trained nursing by well-educated, sensible, sympathetic women, who, having studied and attended medical and surgical lectures, have obtained certificates of competency. It has been said by many leading men in the profession that the services of first-class nurses are, in the treatment of certain diseases, quite as important as the employment of a first-rate physician. Only those whose experience carries them back twenty or twenty-five years can thoroughly realise the enormous improvement since that time in the public and private nursing in this country. But this change has called for many things that cost money. Better nursing demands more clean linen, more baths, better sanitary arrangements, a separate home for nurses when off duty, and a division of the labour in the wards, by the appointment of ward maids for the menial work not connected with the care of the patients. Training schools for nurses employed in the wards and in private houses are now attached to many of our large hospitals, with great advantage to the general public as well as to the patients. All this calls for, and deserves, an increased amount of public support.

Although economy of management is no doubt an important factor in determining the relative merits of hospitals, we must not lay too much stress upon it. A liberal, generous diet, and other little accessories, add greatly to the comfort of a patient, and may in some cases expedite recovery. Again, some hospitals provide everything; while in others the patients have to bring their own tea and sugar and other small articles. The Distribution Committee, when determining the awards, have to take all these circumstances into their consideration, and they should be well weighed by those who claim and have the right to criticise the decisions arrived at.

The Council of the Hospital Sunday Fund are, I think, entitled to claim that the hospitals, and consequently the sick poor of London, have derived benefits from the working of the fund beyond those conferred by the pecuniary awards. I have already stated that the accounts of each institution applying for help have to be carefully examined and compared with those of other institutions doing similar work. This examination is conducted by a small committee of gentlemen of great experience and much technical knowledge, who have no pecuniary interest in any of the institutions applying. The knowledge that this examination will take place operates beyond all doubt very beneficially on the secretaries and the administrative staff of the metropolitan medical charities.

The Council have frequently indulged a hope (which has not been entirely disappointed) that the machinery and statistical information provided at the Mansion House might be utilised by those who, having money to spare for the relief of the sick poor, are desirous of assistance in forming a proper judgment as to the relative defects and merits of our several hospitals, etc. A few large legacies and some smaller gifts have been received and distributed with the general fund, but not to so large an amount as may yet be expected. The difficulties which trustees feel who have to distribute large sums of money among the hospitals may perhaps be best illustrated by a reference to the moneys awarded to the metropolitan medical charities under the wills of the late Mr. Graham in 1887, and Mr. Quinn in 1888. Under the Graham bequest £50,000 was thus awarded: To fourteen general hospitals, £19,500; 34 special hospitals, £26,750; 8 dispensaries, convalescent homes, £3,750. Under the Quinn bequest £28,300 was awarded as follows: Fourteen general hospitals, £18,600; 19 special hospitals, £7,900; 8 dispensaries, etc., £1,800. It will be seen that in the first case the special hospitals, etc., received nearly 50 per cent. in excess of the grant to the general hospitals, while in the second case the general hospitals received more than twice as much as the special. The trustees had a troublesome task assigned to them, and are on the whole to be congratulated on the manner in which they fulfilled it. When we remember the great difficulty of forming precise conclusions on the relative needs of hospitals in the absence of comparative statistical information, we must not be surprised at the different results arrived at.

The sixteen years' work of the Hospital Sunday Fund has not been a career of uninterrupted "peace and progress." The Council had in its early days to encounter difficulties and dangers, requiring careful consideration and great patience, tact, and discretion. During the first few years, the secretaries and managers of some of the hospitals objected strongly to our proceedings, complaining that the Hospital Sunday collection drained the sources of their annual subscriptions, and left them worse off after payment of the award than they were in previous years without it. A few of the managers also strongly protested against the refusal to allow the contributions made by any particular congregation to be entirely handed over to one hospital. The fallacy of the first objection is clearly shown in the returns published in The Hospital newspaper in 1886. These returns cover a period of ten years, from 1876 to 1886, and the figures show that during this period the income of seventy-three hospitals increased 33 per cent., while the expenditure increased only 24 per cent.; that the population increased 21 per cent., while the hospital beds available increased 29 per cent.; and that the in-patients using the hospitals increased 41 per cent., and the out-patients 27 per cent.

It has been suggested that the local interest would be greater if the area of collection were divided into districts and if the money subscribed were apportioned amongst the hospitals in the district in which it was collected; but this system would be very unfair to the hospitals at the East End of London, which are surrounded by the poorest of the sick and suffering. Under the existing plan of distribution, a large share of the money contributed by the wealthy West End congregations finds its way to the relief of the poverty in the East of London. The distribution of the fund has always been a more difficult and anxious task than the collection, for it has been no easy matter to satisfy the friends and managers of more than 150 institutions applying for relief. It was only natural that the several awards should occasionally be objected to and criticised. The objections have always been carefully considered, and the method of distribution has been from time to time modified and improved. The Council believe that the present system is just and equitable, and the best evidence that the subscribing public approve is, I think, to be found in the fact that the amount of the annual collection constantly increases.

I have already referred to the increasing tendency to establish new hospitals, in spite of the very large number of empty beds in existing hospitals; as the majority of these are hospitals for special diseases, it is, I think, useful to consider, as briefly as possible, what are the advantages which special hospitals possess over general hospitals. In speaking of special hospitals, I do not include hospitals for consumption, for contagious diseases, or lying-in hospitals, as the cases taken into these institutions are not often knowingly admitted to general hospitals. The cost per bed for maintenance is admittedly greater in special hospitals; but this ought not to be objected to if the patients are better treated. Whether this is so, however, is a question upon which there is much divergence of opinion. It is, I suppose, agreed that a physician who has given special study to a particular class of disease, or a surgeon who has had great practice in the performance of a particular operation, may be regarded as having more than usual ability and skill for that work; but we then have to ask ourselves whether that special ability and skill cannot be better utilised in a general hospital having special departments than in a special hospital? Few, if any, of the special hospitals, or the small hospitals, have medical schools attached to them, and cannot, therefore, confer the same relative benefit on our enormous metropolitan population that is conferred by general hospitals. Although it may be said that students can resort to special hospitals, few are able to do so. In order to pass their examinations, they must have acquired such a knowledge of medicine and surgery as can only be obtained in general hospitals. After they have passed and have become qualified practitioners, very few are able to spare the time for attendance at hospitals, and these few continue as a rule at the general hospitals. It has been stated by a great authority on the other side of the Atlantic that "Legitimate *specialism* should be recognised only as a superstructure built on a substantial foundation of *generalism*," an opinion which few, I fancy, are prepared to contradict. If I may refer to another and a greater authority, I find that in a recent address Professor Virchow said: "Within twenty-five years the great host of specialties has developed, and it would be vain, anyhow fruitless, to oppose this tendency; but I think I ought to mention it here, and I hope that I shall be certain of approval, when I say that no specialty can flourish which separates itself entirely from the common source of science, that no specialty can develop fruitfully and beneficially, if it does not ever and anon draw from the common fountain, if it does not take the other specialties into account, and if all the specialties do not mutually assist one another." Although Professor Virchow speaks of the great development of specialties in medicine and surgery during the last twenty-five years, it must not be supposed that this division of the work of the physician and surgeon is an idea of modern times; for it will be found that Herodotus, writing in the fifth century B.C., thus describes the practice of physic in Egypt at that time: "Every distinct distemper hath its own physician, who confines himself to the study and care of that alone, and meddles with no other; so that all places are crowded with physicians; for one class hath the care of the eyes, another of the head, another of the teeth, another of the region of the belly, and another of occult distempers."—Lib. ii. c. 84. I was recently looking over a small medical pamphlet, and was much struck by the report of a case of a lady, who was suffering from a supersensitiveness of every part of the body: she had in vain sought relief from numerous specialists, and told a curious story of how each enthusiast treated vigorously and exclusively the organ that belonged to his special domain.

No one can doubt that enormous benefit has been conferred on the public and the profession by the labours and devotion of many specialists, but the balance of competent opinion seems to me to incline to the view that their great talent and ability can be better utilized in the general hospitals, providing proper accommodation and appliances for their work than in the special hospitals.

The pecuniary success of some hospitals supported by voluntary contributions as compared with others, is too often dependent on the tact and smartness of the secretary in the preparation of attractive public festivals, such as fancy fairs, bazaars, dinners, &c., than on the intrinsic merits, as indicated by the good work carried on in the particular institution. This is, perhaps, more the fault of the contributors through such channels than of the secretaries, and it is much to be regretted that subscribers to public charities should allow their contributions to be so largely reduced by the employment of such expensive machinery for collection. The most liberal donors to our public charities are frequently disinclined to take much trouble to satisfy themselves as to the relative merits of the institutions about to be benefited by their generosity. I would strongly urge those who desire to support our medical charities to avail themselves of the statistics collected during the last sixteen years, and carefully preserved at the Mansion House.

Nearly all our large general hospitals require an increase to their annual income, if they are to be maintained in a properly progressive state of efficiency. Unless the necessary funds are provided, some few of them will be compelled to gradually reduce the number of occupied beds, and possibly to close their doors. When we remember the large number of beds in our London hospitals occupied by patients coming from distant parts of the country, suffering from very serious and acute medical and surgical complaints, it would be a national calamity to shut up a single general hospital. A better knowledge of hospital work would increase the public interest in the subject, and this should be stimulated by every legitimate means, until all classes realise the importance and the duty of promoting the prosperity of our large general hospitals, each of us working in the direction in which he feels he can do the greatest amount of good.

